# Idiopathic and structural episodic nonintentional head tremor in dogs: 100 cases (2004‐2022)

**DOI:** 10.1111/jvim.16880

**Published:** 2023-10-18

**Authors:** Theofanis Liatis, Sofie F. M. Bhatti, Magdalena Dyrka, Rodrigo Gutierrez‐Quintana, Rita Gonçalves, Megan Madden, Steven De Decker

**Affiliations:** ^1^ Department of Clinical Science and Services, Royal Veterinary College University of London Hatfield UK; ^2^ Small Animal Department, Small Animal Teaching Hospital, Faculty of Veterinary Medicine Ghent University Merelbeke Belgium; ^3^ Small Animal Hospital, School of Biodiversity One Health and Veterinary Medicine University of Glasgow Glasgow UK; ^4^ Small Animal Teaching Hospital, School of Veterinary Science University of Liverpool Neston UK; ^5^ Hospital for Small Animals, Royal (Dick) School of Veterinary Studies University of Edinburgh Midlothian UK

**Keywords:** bobble‐head doll syndrome, head bobbing, idiopathic head tremor syndrome, secondary episodic head tremor, symptomatic episodic head tremor

## Abstract

**Background:**

Although idiopathic episodic head tremor (IEHT) in dogs is well‐known, little is known about structural brain lesions causing structural episodic head tremor (SEHT).

**Hypothesis/Objectives:**

Describe semiology, magnetic resonance imaging (MRI) findings and outcome of dogs with IEHT or SEHT. We hypothesized that structural lesions affecting the middle cranial fossa or mesencephalic aqueduct could lead to SEHT.

**Animals:**

One hundred dogs with IEHT (n = 71) or SEHT (n = 29).

**Methods:**

Retrospective, multicenter, study of dogs with episodic (nonintentional) head tremor and brain MRI between 2004 and 2022.

**Results:**

Lesions on MRI in SEHT dogs were localized to the middle cranial fossa (15/29), cerebrocortex (3/29), brainstem (2/29), fourth ventricle (1/29) or multifocal (8/29) with thalamus involvement (6/8). Secondary compression of the mesencephalic aqueduct (19/29), third ventricle or interthalamic adhesion or both (14/29) was common. The most common underlying condition in dogs with SEHT was a pituitary mass. Dogs with SEHT were older, had additional neurological signs and were more likely to be euthanized after diagnosis (*P* < .001 for all) compared to IEHT dogs. Two SEHT dogs had only tremor. In IEHT dogs, 8/10 owners reported that the tremor decreased or abated over time (range, 106‐2315 days) without treatment. Tremor remission occurred in SEHT dogs treated for underlying meningoencephalitis.

**Conclusions and Clinical Importance:**

Presence of additional neurological signs and older age may indicate an underlying structural cause for episodic (nonintentional) head tremor involving the mesencephalic aqueduct, third ventricle, interthalamic adhesion or some combination of these. An intracranial structural abnormality cannot be excluded in dogs with normal neurological examination.

**Abbreviations**
BHDS: bobble‐head doll syndrome
CSF: cerebrospinal fluid
FLAIR: fluid attenuation inversion recovery
IEHT: idiopathic episodic head tremor
MRI: magnetic resonance imaging
MUO: meningoencephalitis of unknown origin
SEHT: structural episodic head tremor

## 1 INTRODUCTION

In dogs, episodic (nonintentional) head tremor is an involuntary episodic nonintentional isolated tremor of the head, classified as action‐related postural tremor, which occurs mainly at rest, can usually be terminated by distraction, and can have a vertical (yes‐yes), horizontal (no‐no) or rotational direction.^1^, ^2^, ^3^ Reported occurrence of episodes varies from multiple episodes per day to sporadically or even less frequently than 1 episode every 3‐4 months.^1^ Episodic head tremor is the single clinical sign of a movement disorder called idiopathic episodic head tremor (IEHT) in dogs.^1^ Several breeds have been reported with IEHT,^1^ of which Bulldogs and Dobermans have been associated with a potential breed predisposition, with both familial and sporadic occurrences reported.^4^, ^5^ Although several factors have been proposed,^2^, ^3^, ^6^ the etiology and pathophysiology of IEHT remains unknown. In IEHT, magnetic resonance imaging (MRI) of the brain is normal.^4^, ^5^ Obtaining a diagnosis therefore is based on a combination of compatible clinical signs in a dog of a susceptible breed and absence of signs indicative of epileptic seizures or structural brain disease. In studies of dogs, MRI of the brain often is not considered a strict inclusion criterion for the diagnosis of IEHT,^1^, ^2^, ^5^, ^7^ and the possibility of structural episodic head tremor (SEHT) therefore cannot completely be excluded. However, SEHT has been described in 2 dogs secondary to suprasellar lesions.^8^, ^9^

Episodic (nonintentional) head tremor might share some parallels with bobble‐head doll syndrome (BHDS),^5^ which is a rare movement disorder most commonly affecting children <5 years old, characterized by episodic forward and backward (yes‐yes) or side‐to‐side (no‐no) isolated head movement.^10^ Its frequency is 2‐3 Hz, it disappears with volitional activity, is absent during sleep, and its amplitude typically increases with walking or excitement and temporarily decreases or disappears when the child concentrates or is called.^11^ Bobble‐head doll syndrome has been associated with obstruction of ventricular communication at the level of the third ventricle and mesencephalic aqueduct,^11^, ^12^ such as in suprasellar cysts, third ventricular cysts or mesencephalic aqueductal obstruction.^13^ Other than BHDS, similar phenomenology might be seen in humans with essential tremor, dystonic head tremor, spasmus nutans, Parkinsonian tremor, and epileptic or nonepileptic myoclonus.^11^, ^14^, ^15^ Epileptogenic activity only was found in a single study of dogs with IEHT, and therefore epileptic seizures cannot be completely ruled out. Additionally, episodic vertical head nodding has been described as a part of the phenomenology in juvenile myoclonic epilepsy of Rhodeshian Ridgebacks, however it is usually accompanied by other twitches or progresses to generalized myoclonus.^6^, ^16^

To date, no study has explored the MRI findings in dogs with SEHT. Our aim was to describe the signalment, semiology, MRI findings and outcomes of dogs with IEHT and SEHT. We hypothesized that dogs with middle cranial fossa, including third ventricular, thalamus and interthalamic adhesion, or mesencephalic aqueduct lesions, could develop SEHT.

## 2 MATERIALS AND METHODS

Ours was a retrospective, multicenter, case series study conducted at 5 veterinary teaching hospitals between 2004 and 2022. Ethical approval was granted by the Royal Veterinary College Social Sciences Research Ethical Review Board (URN: SR2021‐0192).

Search terms included: head and tremor, bob, bobbing, nodding, and bobble. Inclusion criteria consisted of (a) complete medical records, (b) clinical features consistent with reported or present episodic or intermittent or transient, nonintentional, isolated head tremor, and (c) MRI of the brain. Exclusion criteria included tremors with an unclear description, permanent tremors, intentional tremors, tremors affecting other parts of the body other than the head, evidence of epileptic seizures, and jerk or shock‐like movements (myoclonus).

Complete medical records consisted of signalment, presenting complaints, clinical, neurological, clinicopathological and MRI findings, final diagnosis, concurrent diseases, treatment and outcome. Cerebrospinal fluid (CSF) analysis, video recording of the tremor, and other test results were evaluated when available. Onset of clinical signs was categorized into hyperacute (<24 hours), acute (1‐7 days), subacute (7‐15 days) and chronic (>15 days).^17^ All clinical and neurological examinations were performed by a board‐certified neurologist or a neurology resident under the direct supervision of a board‐certified neurologist. Magnetic resonance imaging devices used included high‐field or low‐field magnets (Supplementary material). Magnetic resonance imaging sequences performed varied among institutions but always included transverse and sagittal plane T2‐weighted (T2W), fluid attenuation inversion recovery (FLAIR), and T1‐weighted (T1W) pre‐ and postcontrast (gadopentetate dimeglumine, 0.1 mmol/kg IV bolus) images.

**VIDEO 1:** Video footage of the phenotypic characteristics of episodic (nonintentional) head tremor in dogs diagnosed with idiopathic and structural episodic head tremor. Note that episodic head tremor clinically represents the opposite of an intention tremor of the head, because the former usually is discontinued when a goal is set (eg, feeding a treat) whereas the latter is exacerbated when trying to reach the goal.

Dogs were divided into 2 groups based on absence (IEHT) or presence (SEHT) of a structural lesion of the brain on MRI. Functional abnormalities of the brain in the absence of a structural lesion (eg, idiopathic epilepsy) or congenital malformations without associated encephalopathy (eg, Chiari‐like malformation) were not considered structural lesions of the brain.

Follow‐up was achieved at 3 ways: (a) on discharge, (b) by clinical or phone call re‐examination (if available), and (c) by owner questionnaire. The referring veterinarians also were contacted during the follow‐up period. Consistent with the ethical approval of the study, only owners of dogs presumed to be alive were contacted. Owners received an email, which included a consent form and an online questionnaire (Supporting information). The aims of the questionnaire were to gain further insight into the long‐term outcome of the dog from the owner's perspective, understand the current quality of life, and assess the owner's perspective on treatment efficacy and natural history of the disease.

Statistical analysis was performed using standard statistical software (SPSS Statistics 26, IBM Corporation, Armonk, New York). Data were assessed for normal distribution using the Shapiro‐Wilk test for normality. Nonnormally distributed numerical variables were represented as median, interquartile range (IQR) and range. Categorical variables were summarized as counts and percentages. The groups were compared using the Mann‐Whitney *U* test (2 groups) or Kruskal‐Wallis *H* test (>2 groups) in the case of numerical variables, and by the maximum likelihood *G* test or Fisher's exact test (if the expected count in any cell of the contingency table was <5) for categorical variables. Significance level (*α*) was set at .05 and all statistical tests were 2‐tailed. Univariate analysis was performed between variables (sex, neuter status, body weight, age at presentation, age at onset of clinical signs, duration of the tremor before presentation, consciousness during tremor episode, presence of additional neurological signs, presence of spinal hyperesthesia, death, or euthanasia before discharge) and final diagnosis. Variables that were significant in the univariate analysis were further analyzed by multivariable analysis.

## 3 RESULTS

Of the 1812 cases that met the search term criteria, 100 dogs met the inclusion criteria, of which 71 dogs were diagnosed with IEHT based on unremarkable MRI and 29 dogs with SEHT based on lesions identified on MRI of the brain.

### 3.1 Dogs with IEHT

#### 3.1.1 Signalment, history, and presentation

Forty‐two (59.1%) dogs were males and 29 (40.8%) were females, of which a total of 42 (59.1%) were neutered. Median age at onset of the tremor was 1.9 years (range, 1.2 months −11.7 years; IQR, 3 years), and median age at presentation was 2.2 years (range, 3.6 months to 12.2 years; IQR, 3.8 years). Median body weight was 24.6 kg (range, 3.5‐59.4 kg; IQR, 22 kg). The most represented breeds were English Bulldogs (7/71; 9.9%), crossbreeds (6/71; 8.5%), Boxers (5/71; 7%), sighthound‐cross (Lurcher; 5/71; 7%) and Cavalier King Charles Spaniels (5/71; 7%) (Supplementary material). All dogs had episodic (nonintentional) head tremor as a presenting complaint. Additional presenting complaints were epileptic seizures (5/71; 7%) and aggression (4/71; 5.6%; Supplementary material). Mean duration of the tremor occurrence before presentation was 30 days (range, 1‐2880 days; IQR, 111 days). Onset of tremors was acute (14/71; 19.7%), subacute (12/71; 16.7%) or chronic (45/71; 63.4%).

Physical examination abnormalities were found in 16/71 dogs (22.5%) and all of them were unrelated (eg, heart murmur, iris atrophy, degenerative joint disease). Most dogs had normal neurological examination findings (67/71; 94.3%). Neurologic deficits were evident in 4/71 (5.6%) dogs that presented with epileptic seizures, and episodic (nonintentional) head tremor (Video 1) was an incidental finding (Table 1). These were postictal signs such as ataxia (3/71; 4.2%), postural reaction deficits (2/71; 2.8%), symmetric menace response deficits (2/71; 2.8%), tetraparesis (1/71; 1.4%) and disorientation (1/71; 1.4%). Spinal hyperesthesia was evident in 9/71 (12.7%) dogs (Supplementary material).

**TABLE 1:** Episodic head tremor semiology in idiopathic and structural episodic head tremor.

Episodic (nonintentional) head tremor semiology		
	Idiopathic episodic head tremor (IEHT) (n = 71)	Structural episodic head tremor (SEHT) (n = 29)
Distractible to call of name, sound, or volitional movement^a^	Distractible (28/41; 68.3%) Nondistractible (13/41; 31.7%)	Distractible (9/10; 90%) Nondistractible (1/10; 10%)
Direction of episodic head tremor^b^	Horizontal (22/65; 33.8%) Vertical (21/65; 32.3%) Rotatory (7/65; 10.8%) Changing directions (1/65; 1.5%)	Horizontal (4/12; 33.3%) Vertical (7/12; 58.3%) Rotatory (1/12; 8.3%) Changing directions (1/12; 8.3%)
Consciousness during episodic head tremor^c^	Intact consciousness (62/65; 95.4%) Possibly impaired (3/65; 4.6%)	Intact consciousness (23/24; 95.8%) Possibly impaired (1/24; 4.2%)
Episode duration^d^	Median duration 6 minutes (range, 5 seconds to 6 hours)	Median duration 1 minute (range, 30 seconds to 3.5 minutes)
Episode frequency^e^	Daily (from 1 to multiple per day): 19 Weekly: 12 Sporadically (from 1 per month to every 6‐9 months): 10	Daily (from 1 to multiple per day): 9 Weekly: 5
Progression over time regarding its occurrence frequency^f^	Progressive (13/29; 44.8%) Nonprogressive (16/29; 55.1%)	Progressive (10/13; 76.9%) Nonprogressive (3/13; 23.0%)
Reported triggers	Stress (n = 2) Exercise (n = 2) Fish oils, flea treatment, phenobarbital administration, hospitalization, being told off by the owner, fight with another dog, involvement in a car accident, tiredness, playing with a toy, fireworks, whelping, traveling, dietary change (each of them n = 1)	None
Medications used before referral which reportedly decreased episodic head tremor	Phenobarbital (n = 3) Rectal diazepam (n = 1)	None
Medications used before referral which reportedly did not alter episodic head tremor	Imepitoin, meloxicam, propentofylline (n = 2 each) amantadine, methylprednisolone, potassium bromide (n = 1 each)	Phenobarbital, imepitoin, oral diazepam, meloxicam, and gabapentin (n = 1 each)

^a^ Data available for 41 dogs with IEHT and 10 with SEHT.

^b^ Data available for 65 dogs with IEHT and 12 with SEHT.

^c^ Data available for 65 dogs with IEHT and 24 with SEHT.

^d^ Data available for 48 dogs with IEHT and 8 with SEHT.

^e^ Data available for 41 dogs with IEHT and 14 with SEHT.

^f^ Data available for 29 dogs with IEHT and 13 with SEHT.

#### 3.1.2 Diagnostic tests and diagnosis

Magnetic resonance imaging of the brain did not identify any structural abnormality of the brain in any of the dogs. Concomitant findings included intervertebral disc protrusion (3/71; 4.2%). Cerebrospinal fluid (CSF) analysis was performed in 54/71 (74.6%) dogs consisting of sampling from cerebellomedullary cistern (49/54; 92.5%), lumbar cistern (3/54; 5.1%) or both (1/54; 1.9%), all of which findings were unremarkable. Two dogs (2/71; 2.8%) had electroencephalography performed, which was unremarkable (Supplementary material). All dogs were diagnosed with IEHT. Of them, 8 dogs were presented for signs other than tremor, and IEHT was an incidental diagnosis: idiopathic epilepsy (5/71; 7%) and intervertebral disc protrusion (3/71; 4.2%).

#### 3.1.3 Treatment

Most dogs with IEHT were not treated (46/71; 64.8%). The remaining dogs received phenobarbital (9/71; 12.7%), of which 3 were treated for idiopathic epilepsy, gabapentin (5/71; 7%) of which 1 was treated for idiopathic epilepsy, levetiracetam (5/71; 7%), gluten‐free diet (2/71; 2.8%), potassium bromide (2/71; 2.8%), prednisolone (1/71; 1.4%), and a combination of levetiracetam and prednisolone (1/71; 1.4%). All dogs were alive on discharge. Most dogs (60/71; 84.5%) were lost to follow‐up. For dogs that were available for follow‐up (10/71; 14.1%), improvement of episodic (nonintentional) head tremor was seen in 5/11 (45.5%) dogs treated with each of gabapentin, phenobarbital, potassium bromide, levetiracetam or gluten‐free diet. No change of tremor occurrence was observed in dogs treated with phenobarbital (2/71; 2.8%), and each of prednisolone, levetiracetam, or their combination (1/71; 1.4%).

### 3.2 Dogs with SEHT

#### 3.2.1 Signalment, history, and presentation

This group consisted of 16 (55.2%) males and 13 (44.8%) females, of which a total of 22 (75.9%) were neutered. Median age at onset of neurological signs was 6.9 years (range, 6 months −13.7 years; IQR, 6.1 years), and median age at presentation was 6.9 years (range, 6 months to 13.7 years; IQR, 5.7 years). Median body weight at presentation was 19.9 kg (range, 5.8‐38.0 kg; IQR, 16.7 kg). The most represented breeds were French Bulldogs (5/29; 17.2%), crossbreeds (4/29; 13.8%), and Labrador retriever (3/29; 10.3%; Supplementary material). Presenting complaints included lethargy (16/29; 55.1%), circling (8/29; 27.6%), abnormal gait (8/29; 27.6%), anorexia (6/29; 20.7%), generalized tonic‐clonic seizures (5/29; 20.7%), and others (Supplementary material). Episodic head tremor was the most common presenting complaint (26/29; 89.7%) among others, but not usually the main reason for referral. Except for 2 dogs, all dogs had episodic (nonintentional) head tremor in combination with other neurological signs at the time of admission. In the remaining 2 dogs, the tremor was initially the sole clinical sign, but additional neurological signs subsequently developed over 15‐30 days. Mean duration of neurological signs along with the tremor before presentation was 14 days (range, 1‐365 days; IQR, 117 days). Onset of neurological signs along with tremor was acute (13/29; 44.8%), subacute (2/29; 6.9%) or chronic (14/29; 48.3%). Episodic (nonintentional) head tremor of SEHT had features similar to IEHT (Table 1). Accompanying neurological findings included abnormal mentation (21/29; 72.4%), postural reaction deficits (14/29; 48.2%), cranial nerve deficits (13/29; 44.8%), circling (9/29; 31%), paresis (8/29; 27.6%) and other signs (Supplementary material).

#### 3.2.2 Diagnostic tests and diagnosis

**FIGURE 1:**
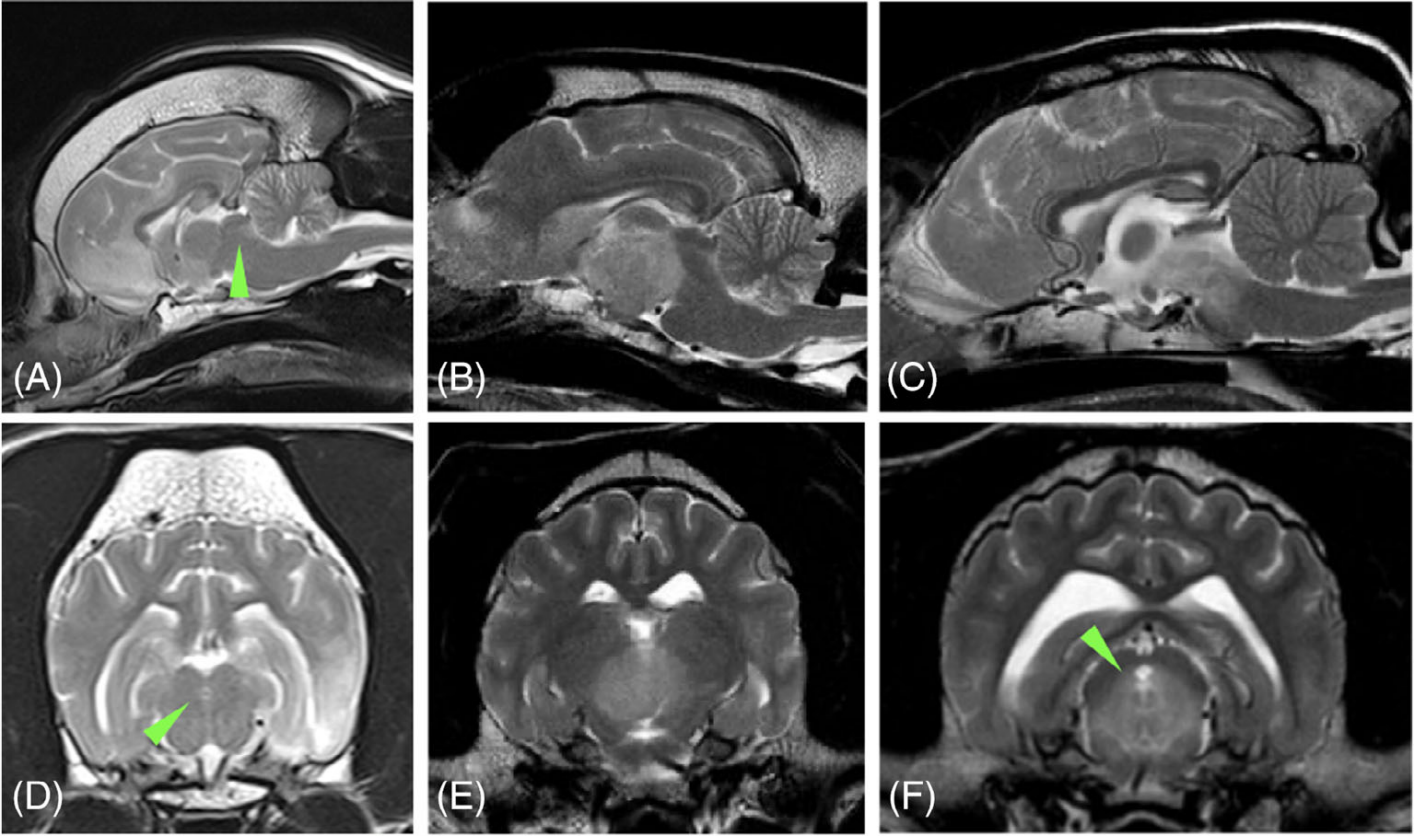
Magnetic resonance imaging of dogs diagnosed with structural episodic head tremor showing mesencephalic aqueduct compression or inflammation. (A and D) T2W sagittal (A) and transverse (D) images of a dog diagnosed with meningoencephalitis of unknown origin showing T2W hyperintensity in the forebrain, including thalamus and the narrowing of the mesencephalic aqueduct (arrowhead) because of compression from the forebrain. (B and E) T2W sagittal (B) and transverse (E) images of a dog diagnosed with a middle cranial fossa mass causing compression of the mesencephalic aqueduct and disappearance of the cerebrospinal fluid signal. (C and F) T2W sagittal (C) and transverse (F) of a dog diagnosed with meningoencephalitis of unknown origin at the level of brainstem without obvious compression of the mesencephalic aqueduct but expansion of the inflammation periaqueductally (arrowhead).

**FIGURE 2:**
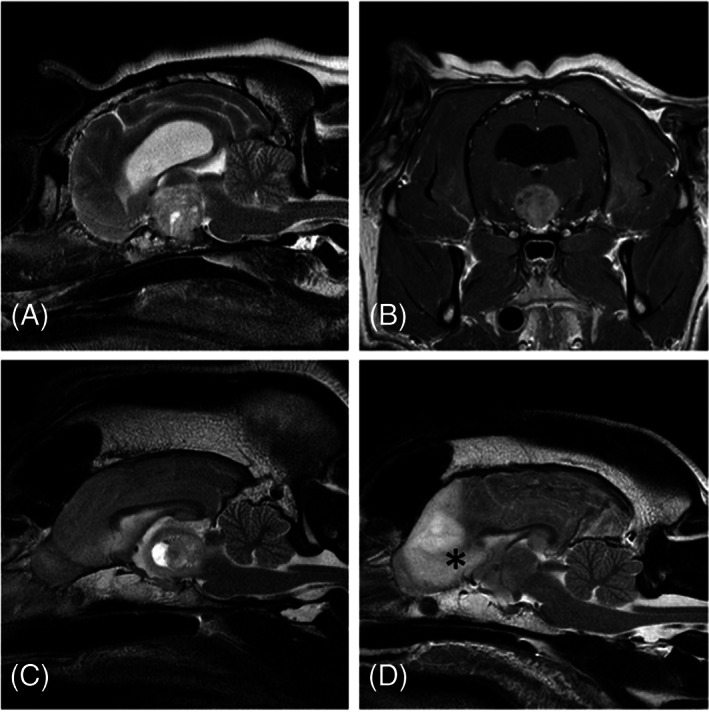
Magnetic resonance imaging of dogs diagnosed with structural episodic head tremor showing third ventricle and interthalamic adhesion compression. (A‐C) T2W sagittal (A), T1W postcontrast transverse (B), T2W sagittal (C) images of different dogs with middle cranial fossa masses compressing the third ventricles and interthalamic adhesion distorting the physiological anatomy. (D) T2W sagittal of a dog with forebrain mass (asterisk) causing secondary compression caudally and distortion of the normal anatomy of the third ventricle.

All dogs underwent MRI of the brain, and of them 2/29 (6.9%) included also the cervical and in 1/29 (3.5%) the complete vertebral column. Lesions found on MRI were localized to middle cranial fossa structures (including pituitary gland, interthalamic adhesion, third ventricle, and thalamus; 15/29; 51.7%), multifocal brain (8/29; 27.6%) of which 6/8 (75%) had thalamic involvement, brainstem (2/29; 6.9%), cerebral cortex (3/29; 10.3%) or fourth ventricle/cerebellum (1/29; 3.5%). More specifically, MRI main findings included middle cranial fossa mass (10/29; 34.5%), T2W/FLAIR hyperintensity of midbrain (9/29; 31%), thalamus (9/29; 31%), pons (7/29; 24.1%) or rostral medulla oblongata (5/29; 17.2%), and cerebral cortex mass (4/29; 13.8%). Of the middle cranial fossa masses, 7/10 were pituitary gland masses, 2/10 extra‐axial sellar and suprasellar masses and 1/10 was a suspected thalamic glioma. Additional findings included mesencephalic aqueduct compression with or without T2W periaqueductal hyperintensity (19/29; 65.5%; Figure 1), or compression or displacement of the third ventricle and interthalamic adhesion (14/29; 48.2%; Figure 2). Cerebellomedullary cisternal CSF analysis was performed in 15/29 (51.7%) dogs and was abnormal in 9/15 (60%) consisting of mixed mononuclear pleocytosis (8/9; 88.9%) and mixed macrophagic and neutrophilic pleocytosis (1/9; 11.1%). None of the dogs had electroencephalography performed. Other diagnostic tests were performed depending on the case (Supplementary material).

All dogs were diagnosed with SEHT. Underlying diseases included middle cranial fossa mass (11/29; 37.9%), forebrain mass (4/29; 13.8%), brainstem mass (confirmed glioma; 1/29; 3.5%), fourth ventricle mass (1/29; 3.5%), complex congenital malformations of the brain (2/29; 6.9%), meningoencephalitis of unknown origin (MUO; 8/29; 27.6%), infectious meningoencephalitis (1/29; 3.5%) and hypertensive encephalopathy (1/29; 3.5%; Table 2).

**TABLE 2:** Underlying diseases in dogs with structural episodic head tremor.

Underlying diseases in dogs with structural episodic head tremor	
Middle cranial fossa mass	11/29 (37.9%)
Pituitary macroadenoma	1/11 (9%)
Pituitary hyperplasia	2/11 (18.1%)
Pars distalis hypophyseal adenoma	1/11 (9%)
Germinoma	1/11 (9%)
Suspected glioma	1/11 (9%)
Other suprasellar mass	3/11 (27.2%)
Forebrain mass	4/29 (13.8%)
Suspected glioma	3/4 (75%)
Suspected meningioma	1/4 (25%)
Brainstem mass	1/29 (3.5%)
Glioma with associated aqueductal compression and third ventricle displacement	
Fourth ventricular mass	1/29 (3.5%)
Fourth ventricular mass with compression of the brainstem and obstructive hydrocephalus (third and fourth ventriculomegaly and pineal recess dilation)	
Meningoencephalitis of unknown origin (MUO)	8/29 (27.6%)
With involvement of the midbrain and associated mesencephalic aqueduct compression	7/8 (75%)
With thalamic involvement	4/8 (50%)
With displacement of the third ventricle	1/8 (12.5%)
With cerebrocortical lesions with secondary mass effect and raised intracranial pressure	1/8 (12.5%)
Infectious meningoencephalitis	1/29 (3.5%)
With multifocal lesions including the thalamus, third ventriculomegaly with pineal recess dilation and aqueductal compression	
Hypertensive encephalopathy	1/29 (3.5%)
With thalamic lesions and associated aqueductal compression	
Complex congenital brain malformation	2/29 (6.9%)
Interthalamic adhesion hypoplasia, septum pellucidum agenesis and ventriculomegaly	1/2 (50%)
Corpus callosum hypoplasia, thalamic myelinolysis and pineal recess dilation	1/2 (50%)

#### 3.2.3 Treatment

Thirteen (13/29; 44.8%) dogs were euthanized at the time of diagnosis without treatment. Seven dogs (7/8; 87.5%) diagnosed with MUO were treated with prednisolone 0.5‐2 mg/kg PO q24h and cytosine arabinoside 200 mg/m^2^ IV or SC every 3‐4 weeks,^18^ whereas the remaining dog was euthanized. Palliative treatment with prednisolone 0.5‐1 mg/kg PO q24h was given to 1 dog with a cerebrocortical mass, 1 dog with a fourth ventricle mass and 2 dogs with middle cranial fossa masses (4/29; 13.8%). Dexamethasone 0.15 mg/kg IV q24h, clindamycin 20 mg/kg IV q12h and trimethoprim‐sulfonamide 15 mg/kg PO q12h were given to 1 dog with infectious meningoencephalitis (1/29; 3.5%). One dog with hypertensive encephalopathy received amlodipine, phenobarbital, clopidogrel, enrofloxacin, omeprazole, and dalteparin sodium (1/29; 3.5%). One dog with suspected pituitary macroadenoma received levetiracetam (1/29; 3.5%). One dog with pituitary hyperplasia received phenobarbital (1/29; 3.5%). One dog with pituitary hyperplasia, hypoadrenocorticism and hypothyroidism received prednisolone and levothyroxine (1/29; 3.5%).

Sixteen (16/29; 55.2%) dogs survived to discharge. Most dogs were lost to long‐term follow‐up (8/16; 50%). Complete remission of the tremor was seen in 7/7 dogs treated for MUO and 1 additional dog was treated for infectious meningoencephalitis.

### 3.3 Questionnaires

In the IEHT group, 28/71 dogs were alive, 34 dogs were dead because of reasons unrelated to IEHT, and for 9/71 no information was available at the time of the study. Questionnaires were sent only to owners of the 28/71 (39.4%) dogs that were still alive at the time of study enrollment. Of them, 10/28 (35.7%) responded. Most owners (8/10; 80%) stated that there was mild or no tremor at the time of the survey. Most owners reported that their dogs were conscious during tremor episodes (9/10; 90%). Episodic head tremor was reported to occur in awake dogs during either daytime or night time (7/10; 70%). The duration of the tremor varied from 10 seconds to 2 hours, with the frequency ranging from 4 episodes per day to 2 episodes per year. Rest (3/10; 30%), stress (2/10; 20%), excitement (2/10; 20%) and gluten treats (1/10; 10%) were reported as potential triggers of an episode. Most owners (8/10; 80%) reported that the tremor had decreased >50% or completely abated since the initial diagnosis over a variable period of time (range, 106‐2315 days) without medical treatment, and that their dogs suffered from anxiety. The quality of life of dogs in this group was characterized as excellent (8/10; 80%; Supplementary material).

In the SEHT group, 6/29 dogs were alive and 23/29 were dead all of which because of the final diagnosis associated with SEHT. Questionnaires were sent to the owners of the 6/29 (20.7%) dogs that were alive. Three owners (3/6) responded. Two owners (2/3; MUO cases) reported abatement of the tremors at the time of the survey (168 and 276 days after the start of treatment), whereas 1 owner (suspected pituitary macroadenoma) reported that tremor frequency was the same since diagnosis. Quality of life was graded as 4/10, 5/10 and 10/10 (1/3 each; Supplementary material).

### 3.4 Statistical analysis

In the univariate analysis, dogs with SEHT were significantly older at the time of presentation (*P* < .001) and at the time of onset of neurological signs (*P* < .001) compared to dogs with IEHT. Dogs presenting with accompanying neurological signs other than the tremor were more likely to have SEHT than IEHT (*P* < .001). Dogs with SEHT were more likely to be euthanized at the time of diagnosis (*P* < .001). After multivariate analysis, only presence of accompanying neurological signs (other than the tremor) in dogs with SEHT remained significant (odds ratio [OR], 191.5; confidence interval [CI], 95%: 19.1, 1913.9; *P* < .001).

## 4 DISCUSSION

Episodic, nonintentional tremors, usually terminated by distraction and isolated to the head, during which consciousness is preserved, have a characteristic phenotype and can be either idiopathic or structural in nature. We confirmed that this tremor can be manifested not only in dogs with IEHT as the only neurological sign in the absence of structural brain abnormality, but also in dogs with accompanying neurological signs as a result of an underlying structural brain abnormality (SEHT). Ours is the first report of episodic (nonintentional) head tremor in dogs as a potential consequence of structural brain disease and to compare the clinical features of dogs with IEHT with those of SEHT.

Episodic (nonintentional) head tremor as the sole clinical sign in younger dogs of overrepresented breeds currently is considered highly suggestive for a benign movement disorder and a diagnosis of IEHT. Intracranial imaging studies therefore often are not strongly recommended in dogs with suspected IEHT. Although the response rate was low (10/28 dogs with IEHT), an interesting result of our study was that most owners (80%) of dogs with IEHT considered their dogs to have excellent quality of life. In agreement with results of previous studies,^1^, ^4^, ^5^, ^7^ the tremor in dogs with IEHT had a tendency to spontaneously abate over time. Although improvement also was seen in some dogs with IEHT that received treatment, it is possible that improvement or resolution of tremors also would have occurred without medical treatment.^1^ The role of medical treatment in dogs with IEHT therefore remains unclear, and the potential benefits and risks of various medications should be carefully considered.

Episodic (nonintentional) head tremor along with accompanying neurological signs and older age were significantly associated with SEHT and underlying structural encephalopathy. However, 2 dogs with SEHT presented initially with only episodic (nonintentional) head tremor, whereas additional neurological signs were manifested within 15‐30 days. An intracranial structural abnormality therefore cannot be excluded in dogs with normal neurological examination findings, and the potential value of additional diagnostic tests should be evaluated in each individual case. Breed (overrepresented breeds with IEHT), age of onset (IEHT occurred in younger dogs, whereas SEHT occurred in older dogs), and presence of accompanying neurological signs (SEHT) should be considered in the clinical assessment of episodic (nonintentional) head tremor in dogs.

The most common diagnosis for dogs with SEHT was a disease of the middle cranial fossa including neoplasia, hyperplasia, inflammation, or other disease affecting CSF flow. All middle cranial fossa, cerebrocortical and brainstem masses in our dogs caused direct or indirect compression or involvement of the thalamus, including third ventricle and interthalamic adhesion, or the mesencephalic aqueduct. Although there might be a difference between their frequency, the phenotype of the tremor and localization of lesions in SEHT might resemble BHDS in humans.^11^, ^12^ In humans, BHDS has been associated with suprasellar arachnoid (44%) or third ventricular (25%) cysts, aqueductal stenosis (14%), malfunctioning shunts (6%), other cysts impinging on the third ventricle (eg, cavum velum interpositum, cavum septum pellucidum cysts; 4%), ventricular tumors impinging on the third ventricle (3%) and communicating hydrocephalus (1%).^11^, ^13^, ^19^ Its pathogenesis is generally unknown, but 1 hypothesis suggests that because of a third ventricular CSF flow imbalance or a compressive mass at that level, an increase of the pressure effect distorts the dorsomedial red nucleus and dentatorubrothalamic pathways.^12^ A ball‐valve mechanism has been suggested as the cause of obstruction of the interventricular foramen, third ventricle dilatation and therefore obstructive hydrocephalus and manifestation of BHDS.^20^ Moreover, compression of the medial thalamus (medial dorsal thalamic nuclei), which includes the somatotopic motor representation of head and neck area in humans, might lead to head and neck tremors.^12^, ^21^ Alternatively, BHDS might be a result of extrapyramidal dysfunction,^22^ because its cessation on volitional activities and its presence during rest suggest possible basal nuclei involvement.^12^ On the other hand, BHDS is usually a lower frequency tremor compared to SEHT of dogs, and it is always a result of structural brain disease,^11^ whereas idiopathic cases have not been reported in humans.^20^

Pituitary mass was 1 of the most common diagnoses in dogs with SEHT in our study. In dogs with pituitary masses, SEHT has not been reported as a typical clinical sign.^23^, ^24^, ^25^, ^26^ Although uncharacterized tremors have been reported in 1 study,^25^ it remains uncertain if those represented SEHT. This observation might be either because of the rarity of the clinical sign, or lack of awareness. Similarly in humans, suprasellar mass lesions with third ventricular dilatation and obstructive hydrocephalus often cause severe medial thalamic compression, and yet BHDS is rare.^11^, ^20^ This discrepancy between human and canine patients might arise from the fact that, in humans, masses arising from within the sella turcica rarely are permitted to attain any considerable size before the patient undergoes surgery, and therefore dorsal expansion of the mass into the third ventricle rarely is encountered. Early signs of pituitary mass in humans are headaches and visual impairment,^27^ which in dogs are difficult to detect.^28^ Therefore, in dogs with pituitary mass, mass effect to the third ventricle and CSF circulation has occurred at the time of diagnosis, whereas in humans such mass effect usually is prevented earlier in the clinical course. Interestingly, BHDS is a consistent finding in third ventricular ependymal or suprasellar arachnoid cysts, and it can be reversible after surgical excision.^20^ Therefore, CSF flow disturbances at the respective region might indicate a more specific etiology of BHDS, but this possibility has not yet been established.

Dogs diagnosed with infectious or immune‐mediated meningoencephalitis usually had a multifocal distribution of lesions with involvement of the midbrain or thalamus being a common finding. Mesencephalic aqueduct compression was seen as displacement or narrowing of the aqueduct or lack of CSF in it, whereas occasionally periaqueductal T2W hyperintensity was evident as part of the inflammatory process. Thalamic lesions were observed as compression, T2W hyperintensity consistent with inflammatory foci, or displacement of the third ventricle and interthalamic adhesion. One dog had cerebrocortical lesions with mass effect, increased intracranial pressure and secondary compression and displacement of the mesencephalic aqueduct. All of these dogs had simultaneous onset of SEHT along with other neurologic signs, localization of the lesion as directly or indirectly affecting the thalamus or mesencephalic aqueduct, and complete remission of SEHT after treatment. The clinical signs of SEHT in these cases potentially could prove to be a useful tool during neuroanatomical localization indicating thalamic or mesencephalic aqueduct involvement.

Limitations of our study included its retrospective nature, lack of video documentation for each case, nonstandardized diagnostic protocols throughout hospitals, lack of electroencephalography for all cases and therefore inability to completely rule out myoclonic epilepsy, limited questionnaire responses, and lack of follow‐up in many cases.

In conclusion, EHT can be present in dogs with normal or abnormal MRI of the brain. Idiopathic episodic head tremor can be present in known breeds, at a younger age, usually is not accompanied by other neurological signs, and MRI of the brain is normal. Structural episodic head tremor is observed in older dogs and is accompanied by other neurological signs prompting pursuit of advanced imaging of the brain to rule out structural brain disease. Structural episodic head tremor in dogs might be associated with lesions affecting the thalamus or the mesencephalic aqueduct, and it might be reversible when the underlying disease can be treated.

## CONFLICT OF INTEREST DECLARATION

Authors declare no conflict of interest.

## OFF‐LABEL ANTIMICROBIAL DECLARATION

Authors declare no off‐label use of antimicrobials.

## INSTITUTIONAL ANIMAL CARE AND USE COMMITTEE (IACUC) OR OTHER APPROVAL DECLARATION

Granted by the Royal Veterinary College Social Sciences Research Ethical Review Board (URN: SR2021‐0192).

## HUMAN ETHICS APPROVAL DECLARATION

Authors declare human ethics approval was not needed for this study.

## Supporting information


**Data S1.** Supporting Information.
