# Supplementary material for: Idiopathic and structural episodic nonintentional head tremor in dogs: 100 cases (2004‐2022)
Source: J Vet Intern Med. 2023 Oct 18;37(6):2301–9. doi: 10.1111/jvim.16880 (PMC10658580; doi:10.1111/jvim.16880)
Supplement: Supplementary file 1 — Data S1. Supporting Information. [file JVIM-37-2301-s001.pdf]

Supplementary material

S1. Materials and methods – Diagnostic Imaging Devices

MRI devices
Philips Intera 1.5-T Pulsar System, Philips Medical Systems, Guildford, UK
1.5T Magnetom, Siemens, UK
Philips Intera 1.5T, Philips Healthcare, Amsterdam, Netherlands;
Airis Mate, Hitachi, Japan, 0.2T
Vet-MR Grande 0.24T, Esaote, Italy

S2. Materials and Methods - Questionnaire for dogs diagnosed with episodic head tremor

(To be completed by the owner)

A. Patient information

Patient Name ___________________
Date ___________________
Breed ___________________
Gender ___________________
Date of birth ___________________

B. Tremor assessment

For the purposes of this questionnaire, head bobbing is defined as involuntary head nodding or tremor or trembling

1. Does your dog have any difficulty on doing his/her daily activities due to his/her tremor? *(select only one)*
   ☐ a. None – no head bobbing at any time
   ☐ b. Mild – mild head bobbing not causing difficulty in performing any activities
   ☐ c. Moderate – head bobbing causes difficulty in performing some activities
   ☐ d. Marked – head bobbing causes difficulty in performing most or all activities
   ☐ e. Severe – head bobbing prevents performing some activities

2. The head bobbing is distractible (that is the tremor can be stopped while calling my dog's name or when distracting her/him or when giving a treat or food bowl or a toy) *(select only one)*
   ☐ Yes
   ☐ No
   ☐ Not sure

   If yes, can you identify any distractive factor? *(choose one or more)*
   ☐ turning head to one side or the other
   ☐ calling dog by his/her name
   ☐ offering him/her food
   ☐ making a sound so that the dog turned his/her head
   ☐ asking her/him to perform a task
   ☐ showing him a toy
   ☐ other – please specify

3. Is the head bobbing of your dog occurring episodically (in episodes, on-off, waxy-wainy)? *(select only one)*
   ☐ Yes
   ☐ No

☐ Not sure

4. During a head bobbing episode is your dog conscious (that is, responsive, alert of your presence or aware of the environment and interacting normally with the environment)? *(select only one)*
   ☐ Yes
   ☐ No
   ☐ Not sure

5. Which is the direction of the head bobbing of your dog? *(select one or more options)*
   ☐ Vertical (YES-YES)
   ☐ Horizonal (NO-NO)
   ☐ Rotational
   ☐ Changes directions – please explain
   ☐ Not sure

6. When does the head bobbing usually occur? *(select one or more options)*
   ☐ At rest – when lying down on sternal (on their chest) recumbency (awake)
   ☐ At rest – when lying down on lateral (on the side) recumbency (awake)
   ☐ At rest – during sleep
   ☐ When waking up from sleep
   ☐ When sitting down
   ☐ While standing
   ☐ When walking
   ☐ Not sure

7. Which time of day is the head bobbing observed? *(select only one)*
   ☐ Evening/night hours
   ☐ Daytime hours
   ☐ Both day and night hours
   ☐ Other – please specify
   ☐ Not sure

8. What is the usual duration of a head bobbing episode?
   (open-ended)

9. What is the frequency of head bobbing episodes? (ie. how many of them do happen in a day, week, month or year?) *(open-ende)*

10. Have you identified any factor(s) which triggers the initiation of head bobbing? *(select only or more)*
    ☐ Stress
    ☐ Excitement
    ☐ Exercise
    ☐ Fear
    ☐ Boredom
    ☐ Thunderstorm phobia
    ☐ Separation anxiety
    ☐ Rest
    ☐ Recent hospitalisation
    ☐ Recent or current illness
    ☐ Other: please explain in detail
    ☐ No triggers have been noticed

11. Has the frequency of head bobbing changed since initial diagnosis? *(select only one)*
☐ No – It is the same as before
☐ Yes – It decreased less than 50%, but still present.
☐ Yes – It decreased more than 50%, but still present.
☐ Yes – It has been completely abated – no head bobbing currently – the head bobbing has resolved.

12. Did your dog have any medication to treat the head bobbing? *(select only one)*
☐ Yes
☐ No
If yes, do you know what was the medication? *(open-ended)*

If yes, do you think the medication helped? *(open-ended)*

If yes, is he/she still on this drug?
☐ Yes
☐ No

C. Quality of life and concurrent diseases

1. How would you characterise the quality of life of your dog regarding his/her head bobbing? (0 – very poor quality of life, 10 – excellent quality of life) *(select only one)*
☐ 0
☐ 1
☐ 2
☐ 3
☐ 4
☐ 5
☐ 6
☐ 7
☐ 8
☐ 9
☐ 10

2. Does your dog have any other concurrent disease? Please state: *(open-ended)*

3. Did your dog develop any epileptic seizures ('fits') after diagnosis of 'head bobbing syndrome'? *(select only one)*
☐ Yes
☐ No
☐ Not sure

4. Do you think your dog has any of the conditions or problems below? *(select only one)*
☐ Separation anxiety
☐ Thunderstorm phobia
☐ Boredom
☐ Aggression
☐ Fear
☐ Nervousness
☐ Hyperactivity
☐ Other – please specify
☐ Not sure

5. Is your dog suffering from any other neurological disease from the below? *(select none, one or more)*
☐ Idiopathic epilepsy

☐ Chiari-like malformation and/or syringomyelia
☐ Fly-catching syndrome
☐None of them

6. Would you like to add or share any information related to the head bobbing of your dog if not already asked in any of the questions? *(open-ended)*

Thank you very much for the participation.

S3. Results - Breeds of dogs diagnosed with idiopathic and structural episodic head tremor

**Breeds**	**n (%)**
**Idiopathic episodic head tremor (n=71)**	
English Bulldog	7 (9.9)
Crossbreed	6 (8.5)
Boxer	5 (7)
Lurcher	5 (7)
CKCS	5 (7)
Cocker Spaniel	4 (5.6)
JRT	4 (5.6)
Labrador	4 (5.6)
Tibetan Terrier	3 (4.2)
Labradoodle	3 (4.2)
Doberman	2 (2.8)
GSD	2 (2.8)
Japanese Akita	2 (2.8)
Staffordshire Bull Terrier	2 (2.8)
Whippet	2 (2.8)
American Bulldog	1 (1.4)
French Bulldog	1 (1.4)
Border Terrier	1 (1.4)
Boston Terrier	1 (1.4)
Alaskan Malamute	1 (1.4)
Cane Corso	1 (1.4)
Dalmatian	1 (1.4)
Cockapoo	1 (1.4)
Flat coated Retriever	1 (1.4)
Irish Terrier	1 (1.4)
Italian Greyhound	1 (1.4)
Lhasa Apso	1 (1.4)
Mastiff	1 (1.4)
Pointer	1 (1.4)
Rottweiler	1 (1.4)
**Structural episodic head tremor (n=29)**	
French Bulldog	5 (17.2)
Crossbreed	4 (13.8)
Labrador	3 (10.7)
Greyhound	2 (6.9)
Dachshund	2 (6.9)

Boxer	2 (6.9)
Boston Terrier	1 (3.5)
CKCS	1 (3.5)
Doberman	1 (3.5)
English Bulldog	1 (3.5)
Fauves De Bretagne - X	1 (3.5)
Flat Coated Retriever	1 (3.5)
Mastiff	1 (3.5)
Miniature Schnauzer	1 (3.5)
Patterdale Terrier	1 (3.5)
Petit Basset Griffon Vendeen	1 (3.5)
Staffordshire Bull Terrier	1 (3.5)

S4. Results – Presenting complaints and neurological findings of dogs diagnosed with idiopathic and structural episodic head tremor

**Idiopathic episodic head tremor (n=71)**	**n (%)**
Presenting complaints	
Episodic head tremor	71 (100)
Generalised tonic-clonic seizures	5 (7)
Aggression	4 (5.6)
Abnormal gait	2 (2.8)
Lethargy	2 (2.8)
Reluctance to exercise	1 (1.4)
Vacant episodes	1 (1.4)
Lameness	1 (1.4)
Body weight gain	1 (1.4)
Senile tremors	1 (1.4)
Dyskinesia/dystonia episode	1 (1.4)
Urinary incontinence	1 (1.4)
Ear scratching	1 (1.4)
Neurological findings	
Episodic head tremor	71 (100)
Spinal hyperaesthesia	9 (12.7)
Ataxia	4 (5.5)
Postural reaction deficits	2 (2.8)
Symmetrical cranial nerve deficits	2 (2.8)
Tetraparesis	1 (1.4)
Disorientation	1 (1.4)
**Structural episodic head tremor (n=29)**	**n (%)**
Presenting complaints	
Episodic head tremor	26 (89.7)
Lethargy	16 (55.1)
Circling	8 (27.6)
Abnormal gait	8 (27.6)
Anorexia	6 (20.7)
Generalised tonic-clonic seizures	5 (17.2)
Urinary incontinence	5 (17.2)
Vacant episodes	4 (13.8)
Head pressing	3 (10.3)
Pacing	3 (10.3)
Diarrhoea	3 (10.3)

Vomiting	3 (10.3)
Stuck in corners	3 (10.3)
BW loss	2 (6.9)
Bumping into objects	2 (6.9)
Feacal incontinence	2 (6.9)
Aggression	2 (6.9)
Easily irritable/hypersensitive	2 (6.9)
Tachypnoea	2 (6.9)
Hemiparesis	2 (6.9)
Low head carriage	2 (3.6)
Head and body turn	2 (3.6)
Head tilt	2 (3.6)
Reluctance to exercise	1 (3.5)
Lip smacking	1 (3.5)
Cough	1 (3.5)
Pyrexia	1 (3.5)
Polyphagia	1 (3.5)
Vocalisations	1 (3.5)
Adipsia	1 (3.5)
Restlessness	1 (3.5)
Neurological findings	
Episodic head tremor	29 (100)
Abnormal mentation	21 (72.4)
Postural reaction deficits	14 (48.2)
Cranial nerve deficits	13 (44.8)
Ataxia	12 (41.4)
Circling	9 (31.0)
Paresis	8 (27.6)
Pleurothotonus	6 (20.7)
Spinal hyperaesthesia	6 (20.7)
Pacing	4 (13.8)
Stuck in corners	4 (13.8)
Head turn	4 (13.8)
Head tilt	3 (10.3)
Head pressing	2 (6.9)
Vacant episodes	2 (6.9)
Generalised hyperaesthesia	1 (3.5)
Plantigrade	1 (3.5)
Bruxism	1 (3.5)

S5. Results – Diagnostic investigations performed in 100 dogs diagnosed with episodic head tremor

Diagnostic tests	IEHT (n=71)	SEHT (n=29)
CBC		
Normal	51 (71.8)	19 (65.5)
Abnormal	10 (14.1)	9 (31.0)
Not done	10 (14.1)	1 (3.5)
Serum biochemistry		
Normal	48 (67.6)	13 (44.8)
Abnormal	12 (16.9)	15 (51.7)
Not done	11 (15.5)	1 (3.5)

Venous blood gas analysis		
Normal	13 (18.3)	6 (20.7)
Abnormal	0 (0)	1 (3.5)
Not done	59 (83.1)	22 (75.9)
Urinalysis		
N1ormal	15 (21.1)	6 (20.7)
Abnormal	2 (2.8)	3 (10.3)
Not done	55 (77.5)	20 (69.0)
MRI		
Head	55 (77.4)	26 (89.7)
Head & whole vertebral column	3 (4.2)	1 (3.5)
Head & cervical vertebral column	11 (15.5)	2 (6.9)
Head & lumbosacral vertebral column	2 (2.8)	0 (0)
CSF analysis		
Normal	53 (74.6)	6 (20.7)
Abnormal	0 (0)	9 (31.0)
Not done	18 (25.3)	14 (48.2)
CSF sample site		
Cerebellomedullary cisternal	49 (92.5)	14 (100)
Lumbar cisternal	3 (5.7)	0 (0)
Both	1 (1.9)	0 (0)
Other tests		
Radiograph thoracic	1	3
Radiograph abdominal	1	1
Radiograph cervical	1	1
Ultrasound abdominal	4	4
Ammonia	6	1
TT4/TSH	4	1
Bile acid stimulation test	18	1
B12 and folate	0	1
Cortisol	2	4
CT thoracic and abdominal	0	3
PT/APTT	0	2
Cytology lymph node	1	1
Cytology liver	0	1
Cytology spleen	0	1
Urine metabolic screen	3	0
Muscle biopsy	0	1
Cardiac troponin I	0	1
ECG	1	2
L2GHA genetic test	1	0
Faecal parasitology	0	1
Urine culture	1	1
LDDST	0	1
Anti-gliadin IgG serology Anti-	1	0
transglutaminase 2 IgA serology	1	0
ACTH stimulation test	2	1
EEG interictal	2	0
Fructosamine	2	0
Anti-AchR antibodies serology	1	0
BAER	1	0
Angiostrongylus serology	1	2
Toxoplasma serology	9	5
Neospora serology	7	4
Distemper PCR in CSF	7	3
Toxoplasma PCR in CSF	7	3

Neospora PCR in CSF	7	2
Ehrlichia PCR in CSF	0	1
Leishmania PCR in blood	0	1
Ehrlichia PCR in blood	0	1
Babesia PCR in blood	0	1
Cryptococcus LAT in blood	0	1
Snaptest 4DX (IDEXX)	0	3
Blood culture	0	1
CSF culture	0	2

S6. Results – Concurrent diseases

Concurrent diseases in dogs diagnosed with idiopathic episodic head tremor and structural episodic head tremor

**Concurrent diagnoses**	**n (%)**
**Idiopathic episodic head tremor (n=71)**	
Supracollicular fluid accumulation	6 (8.5)
Chiari-like malformation & syringomyelia	6 (8.5)
Idiopathic epilepsy	5 (7.0)
Behavioural disorder/anxiety	4 (5.6)
Intervertebral disc protrusion	3 (4.2)
Otitis externa	2 (2.8)
Degenerative joint disease	2 (2.8)
PSOM	2 (2.8)
Dermatitis	2 (2.8)
Gastritis	1 (1.4)
Presbycusis	1 (1.4)
Retinopathy	1 (1.4)
Atlanto-occipital overlap & syringomyelia	1 (1.4)
Paroxysmal dyskinesia	1 (1.4)
Traumatic frontal sinus haematoma	1 (1.4)
Senile tremors	1 (1.4)
Chronic bronchitis	1 (1.4)
Hypothyroidism	1 (1.4)
Absent septum pellucidum	1 (1.4)
**Structural episodic head tremor (n=29)**	
Chiari-like malformation & syringomyelia	2 (6.9)
Subarachnoid diverticulum	2 (6.9)
Degenerative joint disease	2 (6.9)
Focal pneumonia & liver mass	1 (3.5)
Dilated cardiomyopathy	1 (3.5)
Hypothyroidism	1 (3.5)
Hypoadrenocorticism	1 (3.5)
Idiopathic epilepsy	1 (3.5)
Adrenal phaeochromocytoma	1 (3.5)
Empty sella syndrome	1 (3.5)
Intervertebral disc protrusion	1 (3.5)
Multiple vertebral malformations	1 (3.5)
PSOM	1 (3.5)

Otitis externa	1 (3.5)
Supracollicular fluid accumulation	1 (3.5)
Dorsal atlantoaxial dural band	1 (3.5)

S7. Results – Responses to the questionnaire

Questions	Responses	
	IEHT (10/28; 35.6%)	SEHT (6/28; 24.1%)
**B1. Put a mark in the box to rate the severity of your dog's head bobbing within the last 6 months (choose one):**<br>A. None – no head bobbing at any time<br>B. Mild – mild head bobbing not causing difficulty in performing any activities<br>C. Moderate – head bobbing causes difficulty in performing some activities<br>D. Marked – head bobbing causes difficulty in performing most or all activities<br>E. Severe – head bobbing prevents performing some activities	A – 3/10 (30%)<br>B – 5/10 (50%)<br>C – 0/10 (0)<br>D – 1/10 (10%)<br>E – 0 (0)	A – 2/3 (66.7%)<br>B – 0/3 (0)<br>C – 0/3 (0)<br>D – 0/3 (0)<br>E – 1/3 (33.3%)
**B2.1. The head bobbing is distractible (that is the tremor can be stopped while calling my dog's name or when distracting her/him or when giving a treat or food bowl or a toy)**<br>A. Yes<br>B. No<br>C. Not sure	A – 4/10 (40%)<br>B – 5/10 (50%)<br>C – 1/10 (10%)	A – 1/3 (33.3%)<br>B – 2/3 (66.7%)<br>C – 0/3 (0)
**B2.2. If your answer was 'yes' at the previous question, can you identify any distractive factor?**<br>A. Turning head to one side or the other<br>B. Calling dog by his/her name<br>C. Offering him/her food<br>D. Making a sound so that the dog turned his/her head<br>E. Showing him a toy<br>F. Other	A – 1/10 (10%)<br>B – 3/10 (30%)<br>C – 2/10 (20%)<br>D – 2/10 (20%)<br>E – 1/10 (10%)<br>F – 1/10 (10%)	A – 0/3 (0)<br>B – 1/3 (33.3%)<br>C – 1/3 (33.3%)<br>D – 1/3 (33.3%)<br>E – 0/3 (0)<br>F – 0/3 (0)
**B3. Is the head bobbing of your dog occurring episodically (in episodes, on-off, waxy-wainy)?**<br>A. Yes<br>B. No<br>C. Not sure	A – 7/10 (70%)<br>B – 1/10 (10%)<br>C – 2/10 (20%)	A – 1/3 (33.3%)<br>B – 2/3 (66.7%)<br>C – 0/3 (0)
**B4. During a head bobbing episode is your dog conscious (that is, responsive, alert of your presence or aware of the environment and interacting normally with the environment)?**<br>A. Yes<br>B. No<br>C. Not sure	A – 9/10 (90%)<br>B – 0/10 (0)<br>C – 1/10 (10%)	A – 1/3 (33.3%)<br>B – 1/3 (33.3%)<br>C – 1/3 (33.3%)
**B5. Which is the direction of the head bobbing of your dog (Choose one or more options)?**<br>A. Vertical<br>B. Horizontal<br>C. Rotational<br>D. Not sure<br>E. Other	A – 3/10 (30%)<br>B – 6/10 (60%)<br>C – 1/10 (10%)<br>D – 0/10 (0)<br>E – 0/10 (0)	A – 2/3 (66.7%)<br>B – 1/3 (33.3%)<br>C – 0/3 (0)<br>D – 0/3 (0)<br>E – 0/3 (0)
**B6. When does head bobbing usually occur?**	A – 5/10 (50%)	A – 2/3 (66.7%)

A. At rest – when lying down on sternal (on their chest) recumbency (awake)<br>B. At rest – when lying down on lateral (on the side) recumbency (awake)<br>C. At rest – during sleep<br>D. When waking up from sleep<br>E. When sitting down<br>F. While standing<br>G. When walking<br>H. Not sure	B – 4/10 (40%)<br>C – 5/10 (50%)<br>D – 4/10 (40%)<br>E – 2/10 (20%)<br>F – 2/10 (20%)<br>G – 1/10 (10%)<br>H – 1/10 (10%)	B – 0/3 (0)<br>C – 0/3 (0)<br>D – 0/3 (0)<br>E – 1/3 (33.3%)<br>F – 1/3 (33.3%)<br>G – 0/3 (0)<br>H – 0/3 (0)
**B7. Which time of day is the head bobbing observed? (select only one)**<br>A. Evening/night hours<br>B. Daytime hours<br>C. Both day and night hours<br>D. Other – please specify<br>E. Not sure	A – 0/10 (0%)<br>B – 2/10 (20%)<br>C – 7/10 (70%)<br>D – 0/10 (0%)<br>E – 0/10 (0%)	A – 0/3 (0%)<br>B – 0/3 (0%)<br>C – 2/3 (66.7%)<br>D – 0/3 (0%)<br>E – 0/3 (0%)
**B8. What is the usual duration of a head bobbing episode? (open ended)**	range 10 sec - 2 hours	NA
**B9. What is the frequency of head bobbing episodes? (ie. how many of them do happen in a day, week, month or year?) (open-ended)**	from 4 per day - 2 per year	NA
**B10. Have you identified any factor(s) which triggers the initiation of head bobbing? (select only or more)**<br>A. Stress<br>B. Excitement<br>C. Exercise<br>D. Fear<br>E. Boredom<br>F. Thunderstorm phobia<br>G. Separation anxiety<br>H. Rest<br>I. Recent hospitalisation<br>J. Recent or current illness<br>K. Other: please explain in detail<br>L. No triggers have been noticed	A – 2/10 (20%)<br>B – 2/10 (20%)<br>C – 0/10 (0%)<br>D – 0/10 (0%)<br>E – 0/10 (0%)<br>F – 0/10 (0%)<br>G – 0/10 (0%)<br>H – 3/10 (30%)<br>I – 0/10 (0%)<br>J – 0/10 (0%)<br>K – 1/10 (10%) (Gluten treats)<br>L – 2/10 (20%)	A – 0/3 (0%)<br>B – 0/3 (0%)<br>C – 0/3 (0%)<br>D – 0/3 (0%)<br>E – 0/3 (0%)<br>F – 0/3 (0%)<br>G – 0/3 (0%)<br>H – 0/3 (0%))<br>I – 0/3 (0%)<br>J – 0/3 (0%)<br>K – 0/3 (0%)<br>L – 2/3 (66.7%)
**B11. Has the frequency of head bobbing changed since initial diagnosis? (select only one)**<br>A. No – It is the same as before<br>B. Yes – It decreased less than 50%, but still present.<br>C. Yes – It decreased more than 50%, but still present.<br>D. Yes – It has been completely abated – no head bobbing currently – the head bobbing has resolved.	A – 1/10 (10%)<br>B – 1/10 (10%)<br>C –5/10 (50%)<br>D – 3/10 (30%)	A – 1/3 (33.3%)<br>B – 0/3 (0%)<br>C – 0/3 (0%)<br>D – 2/3 (66.7%)
**B12. Did your dog have any medication to treat the head bobbing? (select only one)**<br>A. Yes<br>B. No<br>If yes, do you know what was the medication? (open-ended)<br>If yes, do you think the medication helped? (open-ended)<br>If yes, is he/she still on this drug?	A – 2/10 (20%)<br>B – 6/10 (60%)<br>Medications:<br>-Levetiracetam (n=1) did not help.<br>-Phenobarbital & diazepam (n=1) did not help.<br>-None of dog on these drugs still.	A – 1/3 (33.3%)<br>B – 2/3 (66.7%)<br>Medications:<br>-Chemotherapy & steroids (n=1) did help.<br>-Still on treatment.

a. Yes<br>b. No		
**C1. How would you characterise the quality of life of your dog regarding his/her head bobbing?<br>(0 – very poor quality of life, 10 – excellent quality of life) (select only one)**<br>A. 0<br>B. 1<br>C. 2<br>D. 3<br>E. 4<br>F. 5<br>G. 6<br>H. 7<br>I. 8<br>J. 9<br>K. 10	A – 0/10 (0%)<br>B – 0/10 (0%)<br>C – 0/10 (0%)<br>D – 0/10 (0%)<br>E – 0/10 (0%)<br>F – 0/10 (0%)<br>G – 0/10 (0%)<br>H – 0/10 (30%)<br>I – 1/10 (10%)<br>J – 1/10 (10%)<br>K – 8/10 (80%)	A – 0/3 (0%)<br>B – 0/3 (0%)<br>C – 0/3 (0%)<br>D – 0/3 (0%)<br>E – 1/3 (33.3%)<br>F – 1/3 (33.3%)<br>G – 0/3 (0%)<br>H – 0/3 (0%))<br>I – 0/3 (0%)<br>J – 0/3 (0%)<br>K – 1/3 (33%)
**C2. Does your dog have any other concurrent disease? Please state: (open-ended)**	-Bladder infection (n=1)<br>-Allergies (n=1)	-Pituitary mass (n=1)<br>-MUO (n=1)
**C3. Did your dog develop any epileptic seizures (‘fits’) after diagnosis of ‘head bobbing syndrome’? (select only one)**<br>A. Yes<br>B. No<br>C. Not sure	A – 0/10 (0%)<br>B – 9/10 (9%)<br>C – 0/10 (0%)	A – 1/3 (33.3%)<br>B – 2/3 (66.7%)<br>C – 0/3 (0%)
**C4. Do you think your dog has any of the conditions or problems below? (select only one)**<br>A. Separation anxiety<br>B. Thunderstorm phobia<br>C. Boredom<br>D. Aggression<br>E. Fear<br>F. Nervousness<br>G. Hyperactivity<br>H. Other – please specify<br>I. Not sure	A – 4/10 (40%)<br>B – 4/10 (40%)<br>C – 0/10 (0%)<br>D – 0/10 (0%)<br>E – 4/10 (4%)<br>F – 8/10 (80%)<br>G – 2/10 (20%)<br>H – 1/10 (10%)<br>I – 0/10 (0%)	A – 0/3 (0%)<br>B – 0/3 (0%)<br>C – 0/3 (0%)<br>D – 0/3 (0%)<br>E – 0/3 (0%)<br>F – 0/3 (0%)<br>G – 0/3 (0%)<br>H – 2/3 (66.7%))<br>I – 0/3 (0%)
**C5. Is your dog suffering from any other neurological disease from the below? (select none, one or more)**<br>A. Idiopathic epilepsy<br>B. Chiari-like malformation and/or syringomyelia<br>C. Fly-catching syndrome<br>D. None of them	A – 1/10 (10%)<br>B – 1/10 (10%)<br>C – 0/10 (0%)<br>D – 6/10 (60%)	A – 1/3 (33.3%)<br>B – 0/3 (0%)<br>C – 0/3 (0%)<br>D – 1/3 (33.3%)
**C6. Would you like to add or share any information related to the head bobbing of your dog if not already asked in any of the questions? (open-ended)**	NA	NA
